# Artificial MicroRNA-Based Specific Gene Silencing of Grain Hardness Genes in Polyploid Cereals Appeared to Be Not Stable Over Transgenic Plant Generations

**DOI:** 10.3389/fpls.2016.02017

**Published:** 2017-01-09

**Authors:** Sebastian Gasparis, Maciej Kała, Mateusz Przyborowski, Waclaw Orczyk, Anna Nadolska-Orczyk

**Affiliations:** ^1^Department of Functional Genomics, Plant Breeding and Acclimatization Institute (IHAR) – National Research InstituteBlonie, Poland; ^2^Department of Genetic Engineering, Plant Breeding and Acclimatization Institute (IHAR) – National Research InstituteBlonie, Poland

**Keywords:** polyploid cereals, amiRNA, siRNA, RNAi, wheat, triticale, grain hardness, *Pin* genes

## Abstract

Gene silencing by RNA interference is a particularly important tool in the study of gene function in polyploid cereal species for which the collections of natural or induced mutants are very limited. Previously we have been testing small interfering RNA-based approach of gene silencing in wheat and triticale. In this research, artificial microRNAs (amiRs) were studied in the same species and the same target genes to compare effectiveness of both gene silencing pathways. amiR cassettes were designed to silence *Puroindoline a* (*Pina*) and *Puroindoline b* (*Pinb*) hardness genes in wheat and their orthologues *Secaloindoline a* (*Sina*) and *Secaloindoline b* (*Sinb*) genes in triticale. Each of the two cassettes contained 21 nt microRNA (miR) precursor derived from conserved regions of *Pina*/*Sina* or *Pinb*/*Sinb* genes, respectively. Transgenic plants were obtained with high efficiency in two cultivars of wheat and one cultivar of triticale after using the *Pinb*-derived amiR vector for silencing of *Pinb* or *Sinb*, respectively. Lack of transgenic plants in wheat or very low transformation efficiency in triticale was observed using the *Pina*-derived amiR cassette, despite large numbers of embryos attempted. Silencing of *Pinb* in wheat and *Sinb* in triticale was highly efficient in the T_1_ generation. The transcript level of *Pinb* in wheat was reduced up to 92% and *Sinb* in triticale was reduced up to 98%. Moreover, intended silencing of *Pinb*/*Sinb* with *Pinb*-derived amiR cassette was highly correlated with simultaneous silencing of *Pina*/*Sina* in the same transgenic plants. High downregulation of *Pinb*/*Pina* genes in T_1_ plants of wheat and *Sinb*/*Sina* genes in T_1_ plants of triticale was associated with strong expression of *Pinb*-derived amiR. Silencing of the target genes correlated with increased grain hardness in both species. Total protein content in the grains of transgenic wheat was significantly lower. Although, the *Pinb*-derived amiR cassette was stably inherited in the T_2_ generation of wheat and triticale the silencing effect including strongly decreased expression of silenced genes as well as strong expression of *Pinb*-derived amiR was not transmitted. Advantages and disadvantages of posttranscriptional silencing of target genes by means of amiR and siRNA-based approaches in polyploid cereals are discussed.

## Introduction

Common wheat (*Triticum* *aestivum* L.) is the world’s third, after corn and rice, major cereal crop and an important human food source (Tester and Langridge, 2010). Triticale (x *Triticosecale* Wittmack) is a hybrid of wheat (*Triticum*) and rye (*Secale*) with higher yield potential and disease, and environmental tolerance but lower grain quality than wheat. In 2014, triticale was cultivated in 37 countries across the world (FAO Statistics Division, 2016). Genomes of these Triticeae species are allohexaploid, composed of three diploid genomes (2*n* = 6*x* = 42), differing for one of them (AABBDD for wheat and AABBRR for triticale). The genome size, which is 17 Gbp for wheat (Bennett and Smith, 1976), and its complexity in both species represent an important limitation in collecting mutants of many important traits and analysis of gene function.

Cereal grain hardness is an important agronomic trait that influences quality of the flour and properties of the end-products. The soft grain phenotype in wheat is determined by wild-type alleles of *Pina* and *Pinb* genes and any mutation in either or both *Pin* gene(s) results in more or less harder grains (reviewed in Bhave and Morris, 2008; Nadolska-Orczyk et al., 2009; Pauly et al., 2013). Consistent with the above is reported by Gasparis et al. (2011) increased grain hardness after siRNA-mediated silencing of the *Pin* genes. *Sina* and *Sinb* genes of hexaploid triticale are orthologs of *Pin* genes in hexaploid wheat. The influence of these genes on grain hardness in triticale is expected to be similar, however, this has not been confirmed (Gasparis et al., 2013).

Gene silencing by RNA interference (RNAi) is a powerful technology of reverse genetics to characterize gene function and to obtain plants with desired characteristics, i.e., enhancing crop yield/productivity, quality and resistance to biotic and abiotic stresses. The technology is based on the natural processes of regulation of gene expression by small RNAs (sRNAs) including microRNAs (miRNAs) and short interfering RNAs (siRNAs) (Ossowski et al., 2008; Axtell, 2013; Rogers and Chen, 2013). These sRNAs, mostly 21–24 nt in length, direct sequence-specific gene regulation originally induced by double stranded RNA (dsRNA), resulting in inhibition of transcription or translation (Hamilton and Baulcombe, 1999; Brodersen and Voinnet, 2006; Borges and Martienssen, 2015). Gene silencing by siRNA, termed in plants as post-transcriptional gene silencing (PTGS; van der Krol et al., 1990) has been widely used since the late 1990s (Waterhouse and Helliwell, 2003; Baulcombe, 2004). The technology became a method of choice for analysis of gene function, especially in polyploid species (Lawrence and Pikaard, 2003; Travella et al., 2006; reviewed by Fu et al., 2007; Gasparis et al., 2011, 2013). The resulted phenotypes were found to be stable inherited through generations.

microRNAs are key regulators of important plant processes, such as growth, development, and response to various stresses (Martin et al., 2010; Ameres and Zamore, 2013). In wheat miRNAs and their targets play a complex role in regulation of grain development (Li et al., 2015). These sRNAs in plants were identified in 2002 by different groups (Llave et al., 2002; Mette et al., 2002; Park et al., 2002; Reinhart et al., 2002). miRNA differs from siRNA in their precursor structures, origin and biogenesis, modes of action, and cellular and developmental functions (Carthew and Sontheimer, 2009; Ghildiyal and Zamore, 2009; Axtell, 2013; Kamthan et al., 2015). The natural mechanism of gene regulation by miRNA was used as base to design experimental tool termed artificial microRNA (amiR) (Ossowski et al., 2008; Tiwari et al., 2014) or miRNA-induced gene silencing (MIGS) (Felippes et al., 2012). In contrast to siRNAs, miRNAs are produced from long, single-stranded RNA molecules exhibiting highly specific stem–loop structures (Chen, 2009). Silencing cassettes of amiRNA are designed by replacing the mature 21 nt miRNA sequences within pre-miRNA with 21 nt long fragment derived from the target gene. Integrated with the genome amiRNA is expressed and the resultant transcript is subsequently processed via small RNA biogenesis. Finally, the active amiRNA molecule triggers silencing of the target gene in a manner similar to miRNAs. amiRNA-based strategies are regarded to be more efficient due to their higher specificity and fewer undesirable off-target effects (Schwab et al., 2006; Meyers et al., 2008; Warthmann et al., 2008) compared to siRNA (Xu et al., 2006). Artificial miRNA was applied to silence diverse groups of genes in different species (Alvarez et al., 2006; reviewed by Tiwari et al., 2014 and Kamthan et al., 2015). Similar to miRNAs, amiRNAs might regulate gene expression at the transcriptional (TGS) or posttranscriptional level.

There are several reports on gene silencing by amiRNA in monocot species. Silencing of endogenous genes in rice was successful, with amiRNAs having been expressed in the OsMIR528 precursor (Warthmann et al., 2008; Butardo et al., 2011; Chen et al., 2012). In wheat, there is only one article on using amiRNA approach to obtain plants resistant to *Wheat streak mosaic virus* (*WSMV*) (Fahim et al., 2012). The strategy was to incorporate five amiRNAs within one polycistronic amiRNA precursor. Resistance was assessed over two generations. The same polycistronic type of vector, which was based on a barley miRNA precursor backbone and targeted different conservative sequences of *Wheat dwarf virus* (*WDV*), conferred barley resistance to the virus at low temperature (Kis et al., 2016). Recent research indicated that synthetic/artificial trans-acting siRNAs (syn-tasiRNAs) carrying multiple virus resistance cassettes might be more effective than amiRNA for enhanced antiviral defense (Carbonell et al., 2016; Chen et al., 2016).

In our previous research, we have tested silencing of *Pina* and *Pinb* hardness genes in wheat (Gasparis et al., 2011) and their orthologs, *Sina* and *Sinb* genes in triticale (Gasparis et al., 2013) by siRNA strategy, using the hairpin type of cassette. The transcript level of the genes was reduced by over 80% in T_1_, and over 90% in T_2_ up to T_4_, and was stable through generations. Moreover, this RNA-mediated silencing of one of the *Pin* or *Sin* genes simultaneously decreased the expression of the second *Pin* or *Sin*. The reduction of transcript levels of both genes resulted in a significant decrease or lack of both puroindoline and secaloindoline proteins and increased grain hardness in wheat. In contrast to the results obtained with the *Pin* genes, the decreased expression of *Sin* genes and lower level of secaloindoline proteins did not affect grain hardness.

In this research, we applied artificial miRNA to silence *Pina*/*Sina* and *Pinb*/*Sinb* grain hardness genes of two hexaploid Triticeae species, wheat and triticale, and to compare the effect of silencing with siRNA in the PTGS pathway. The same research model, i.e., grain hardness genes, and the same cultivars of two polyploid cereal species were used. amiRNAs were designed based on conserved precursor miRNA of wheat, *Tae-miR164*. The very high expression of *Pinb*-derived amiR in the T_1_ generation resulted in strongly decreased expression of *Pinb* and *Sinb* genes in both species, which was correlated with reduced protein level and significantly increased grain hardness. The silencing of one *Pinb* or *Sinb* gene simultaneously influenced silencing of their corresponding paralogue *Pina* or *Sina*. While *Pinb*-derived amiR silencing cassette was stably inherited, the strong effect of silencing was not transmitted to the T_2_ generation and *Pinb*-derived amiR was not expressed in both species. To our knowledge, this is the first report on the effect of silencing by amiRNAs of a structural, endogenous genes in polyploid cereals implicating a non-inherited mode of silencing.

## Materials and Methods

### Plant Material

Two cultivars of Polish spring wheat, Kontesa, and Torka (*Triticum aestivum* L), and one cultivar of spring triticale, Wanad (x *Triticosecale* Wittmack), were used in the experiments. Wild type, donor plants for transformation experiments as well as transgenic plants were grown under controlled conditions of long day (16 h light) as described by Gasparis et al. (2013).

### AmiRNA Design and Cloning, and Vector Construction

Artificial microRNA cassettes for silencing *Pina* and *Pinb* genes in wheat, and *Sina* and *Sinb* genes in triticale, were constructed based on precursor microRNA of wheat Tae-miR164 (Mirbase.org: MI0006173). Primers for amplification and sequencing of precursor Tae-miR164 were designed based on the EST sequence (access number CA704421). The sequences of the primers were: forward 5′-AGGTGGAGAAGCAGGGCACGT-3′ and reverse 5′- TATCACGCAGAGCTGACACCACAA-3′. The primers were used to amplify specific precursor Tae-miR164 in Kontesa and Torka cultivars. In the next step, native fragments of microRNA of 21 bp in precursor Tae-miR164 were replaced with the sequence complementary to the *Pin* and *Sin* genes. The 21 bp sequences of microRNA for these genes were designed based on the Web MicroRNA Designer platform (WMD3^1^) (Ossowski et al., 2008). The sequences were: (1) for *Pina*-derived amiR (ami-Pa): 5′-UGAAAUCCGAAGAUGCCATCG-3′ complementary to the sequence of allele *Pina-D1a* in the region of 321–341 and (2) for *Pinb*-derived amiR (ami-Pb): 5′-UGUUUGAAUACCUCACCUAGC-3′ complementary to the sequence of allele *Pinb-D1c* in the region of 348–368. Precursor microRNA was cloned into the pGEM-T (Promega) vector and used as a template for multiple PCR amplification to insert designed amiRNA into the precursor. Primers used in these reactions are as follows:

(a) Primers for ami-Pa amplification:
  1. ami_pa1 5′-ggTGAAATCCGAAGATGCCATCGttcatttccaggtcgct-3′
  2. ami_pa2 5′-aaCGATGGCATCTTCGGATTTCAccaatcccgcggccatg-3′
  3. ami_pa3 5′-caCGCTGGCATCTTCGGATTTCAccaatcactagtgcggc-3′
  4. ami_pa4 5′-ggTGAAATCCGAAGATGCCAGCGtgcatgggccggctgcc-3′
    pgt_A 5′-actcactatagggcgaattg-3′
    pgt_B 5′-actcaagctatgcatccaac-3′
(b) Primers for ami-Pb amplification:
  1. ami_pb1 5′-ggTGTTTGAATACCTCACCTAGCttcatttccaggtcgct-3′
  2. ami_pb2 5′-aaGCTAGGTGAGGTATTCAAACAccaatcccgcggccatg-3′
  3. ami_pb3 5′-caGCCAGGTGAGGTATTCAAACAccaatcactagtgcggc-3′
  4. ami_pb4 5′-ggTGTTTGAATACCTCACCTGGCtgcatgggccggctgcc-3′
    pgt_A 5′- actcactatagggcgaattg-3′
    pgt_B 5′-actcaagctatgcatccaac-3′

The order of the amplification procedure was: (1) pgt_A and ami_pa2 primers; (2) ami_pa3 and pgt_B primers; (3) ami_pa1 and ami_pa4 primers, and (4) products of amplification from 1 to 3 reactions plus pgt_A, pgt_B primers.

The cassettes containing ami-Pa and ami-Pb were cloned into the pBract214 overexpression vector^2^. The vector is compatible with the Gateway cloning system. In the first step, the cassettes were amplified using: ami_pa5 5′-GGTGAAATCCGAAGATGCCATCGTT-3′, ami_pa6 5′-GATTGGTGAAATCCGAA-3′, and ami_pb5 with: 5′-GGTGTTTGAATACCTCACCTAGCTT-3′, ami_pb6 5′-GATTGGTGTTTGAATAC-3′, respectively and cloned into the entry vector pCR8/GW/TOPO (Invitrogen). In the next step, the cassettes were cloned to the destination pBract214 vector in the Gateway reaction. The presence of the amiRNA in the vectors was verified by restriction analysis and sequencing. The vectors were electroporated into the AGL1 strain of *Agrobacterium tumefaciens* and used for transformation.

### *Agrobacterium*-Mediated Transformation and PCR Testing

*Agrobacterium*-mediated transformation experiments were performed according to our previously described protocols for wheat (Przetakiewicz et al., 2003, 2004) and for triticale (Nadolska-Orczyk et al., 2005). Putative transgenic plants were regenerated and selected on modified MS media containing 20 mg l^-1^ of hygromycin as a selectable agent.

Genomic DNA was isolated from young leaves of 5 days seedlings according to the modified CTAB procedure (Murray and Thompson, 1980). The PCR was carried out in a 25 μl reaction mixture using Platinum Taq DNA Polymerase (Invitrogen) and 120 ng of template DNA. The reaction was run using the following program: initial denaturation step at 94°C for 2 min, 35 cycles of amplification at 94°C for 30 s, 65°C for 30 s, 72°C for 30 s with a final extension step at 72°C for 5 min.

The wheat and triticale plants transformed with *Pina*-derived amiR and *Pinb*-derived amiR vectors were tested with two pairs of specific primers amplifying a fragment of the *hpt* selection gene. The sequences of the primers for the first pair were: pBr_hyg-F2 5′-GACGGCAATTTCGATGATG-3′ and pBr_hyg-R2 5′-CCGGTCGGCATCTACTCTAT-3′, and amplified fragment of 205 bp. The sequences of the primers for the second pair were: pBr_hygF4 5′-ATGACGCACAATCCCACTATCCT-3′ and pBr_hygR4 5′-AGTTCGGTTTCAGGCAGGTCTT-3′, and amplified fragment of 405 bp.

Putative transgenic T_0_ plants were tested with each of the two pairs of specific primers. In case if one PCR test was positive and the second one was negative the third one was applied with one of these two pairs of primers. Only PCR positive (PCR+) plants were grown for further testing. The same PCR tests were applied for eight randomly selected T_2_ progeny from one T_1_ transgenic parent. Segregation rates were estimated based on PCR+/PCR- plants. Non-transgenic, *in vitro* plants as well as null segregants were used as a control.

### RNA Isolation and cDNA Synthesis

Total RNA was isolated from immature kernels at 26 DAP (days after pollination) using the modified SDS extraction method of Prescott and Martin (1986). An additional extraction step was performed using TRI-Reagent (Sigma-Aldrich) according to the manufacturer’s protocol. The RNA samples were digested with DNAse I recombinant (Roche). The first-strand cDNA was synthesized from 1 μg of RNA using the RevertAid First Strand cDNA Synthesis Kit (Thermo Scientific) according to the manufacturer’s instructions.

### Quantitative Real-Time qRT-PCR

The relative expression level of *Pin* genes in wheat and *Sin* genes in triticale was analyzed in 26 DAP samples of transgenic and control plants. The sequences of the primers were as follows: for *Pina* and *Sina*, qPinA1 (forward) 5′-CTCATAGGACTGCTTGCTCTGGTAG-3′, qPinA2 (reverse) 5′-GATTGACCCCTGGATGATGTTG-3′; for *Pinb* and *Sinb*, qPinB1 (forward) 5′-AATGAAGTTGGCGGAGGAGGTG-3′, qPinB2 (reverse) 5′-ATACCTCACCTCGCCAAATGCC-3′; and for 18S rRNA, 18S F (forward) 5′-GTGACGGGTGACGGAGAATT-3′, 18S R (reverse) 5′-GACACTAATGCGCCCGGTAT-3′. The reaction was carried out in a 15 μl mixture containing 1× Hot FIREPol EvaGreen qPCR Mix (Solis BioDyne), 0.4 μM of each primer, and 1 μl of the template cDNA. The following temperature profile was used: an initial denaturation step of 95°C for 15 min, 50 cycles of amplification at 95°C for 20 s, 58°C for 15 s, and 72°C for 20 s, and a melting curve profile of 72–95°C with the temperature rising 1°C at each step and continuous fluorescence measurement. Three replicates of each sample were used in the reaction. The relative expression level of *Pin* and *Sin* genes was calculated by the standard curve method using 18S rRNA as a normalizer. The non-transgenic control plant was used as a calibrator sample with the expression level set to 1. The normalized values of the tested samples are expressed as x-fold of 1.

### Identification of amiRNA in Transgenic Plants

Low molecular weight RNA containing the microRNA fraction was separated from total RNA using polyethylene glycol (PEG) as described by Goto et al. (2003). The artificial *Pinb*-derived miR was detected by stem-loop RT-PCR miRNA assay as described by Varkonyi-Gasic et al. (2007). Two hundred and fifty nanograms of RNA from each sample and 0.05 μM of RT primer were used for the reverse transcription reaction with Maxima reverse transcriptase (Thermo Scientific). The loop region of RT primers contained a sequence complementary to UPL probe #21 (highlighted in bold). Primers for endogenous Tae-miR164 were used in the positive control sample. The sequences for RT and qPCR primers were: for ami-Pb: RT primer 5′-GTTGGCTCTGGTGCAGGGTCCGAGGTATTCGCAC**CAGAGCCA**ACGCTAGG-3′ and forward primer 5′- GGTGTTTGAATACCTCACCTAGC -3′ and reverse primer 5′-GTGCAGGGTCCGAGGT-3′; for Tae-miR164: RT primer 5′-GTTGGCTCTGGTGCAGGGTCCGAGGTATTCGCAC**CAGAGCCA**ACTGCACG-3′, forward primer 5′-TGGAGAAGCAGGGCACGTGCA-3′ and reverse primer 5′-GTGCAGGGTCCGAGGT-3′; and for U6 RNA: forward primer 5′-CTTCGGGGACATCCGATAAA-3′ and reverse primer 5′-GACCATTTCTCGATTTGTGC-3′.

Samples were first incubated at 65°C for 5 min, and the reverse transcription reaction was performed using the following program: 94°C for 2 min followed by 40 cycles of 94°C for 15 s and 60°C for 1 min. The qPCR reaction was carried out in a 20 μl reaction mixture containing 10 μl of TaqMan Fast Advanced Master Mix (Applied Biosystems), 2 μl of cDNA sample, 0.4 μM of each primer and 0.25 μl of UPL probe #21 (Roche). The reaction was run in a RotorGene Q thermal cycler (Qiagen) using the following program: initial denaturation step at 95°C for 2 min followed by 55 cycles of amplification at 95°C for 5 s, 60°C for 15 s, and 72°C for 5 s. U6 RNA was used as a reference gene. A series of dilutions of amplified amiRNA and U6 RNA was used in the first qPCR reaction to estimate the reaction efficiency. The relative level of amiRNA was calculated using the Pfaﬄ method (Pfaﬄ, 2001). The sample with the highest Ct value was used as a calibrator. The level of amiR was determined as x-fold higher compared to the calibrator sample, which was the sample with the lowest detectable amiR level set to 1.0.

### Extraction of Proteins and SDS-PAGE

Friabilin (complex of *puroindoline a* and *puroindoline b*) was extracted from the surfaces of the starch granules using the method of Bettge et al. (1995) with some modifications. To obtain 50 mg of wheat flour, 100–150 mg of kernels were ground in a mortar. After being transferred to a 2 ml microfuge tube containing 0.6 ml of 0.1 M NaCl, the samples were incubated at room temperature for 30 min in a thermoshaker. The aqueous starch suspension was transferred to a new 2 ml microfuge tube and centrifuged at 15 000 × *g* for 1 min. The pellet was washed with 1 ml of water. Using a pipette tip, the gluten and bran residues were formed into a ball and removed. The starch pellet was resuspended in 1 ml of 80% CsCl and centrifuged at 15 000 × *g* for 1 min. The CsCl solution was then removed from the tube, and the sample was washed three times with 1 ml of water and once with 0.6 ml of acetone. The pellet was left to dry and the tubes were weighed to determine the amount of starch. Next, the starch pellets were suspended in 150 μl of 1 M NaCl and 150 μl of isopropanol. After incubation for 45 min at 45°C, the samples were centrifuged at 15 000 × *g* for 1 min. The supernatant was transferred to fresh microfuge tubes and 150 μl of cold acetone was added, and samples were stored overnight at -20°C. Next, the samples were centrifuged at 15 000 × *g* for 1 min and transferred to fresh microfuge tubes. This fraction was precipitated for 2 h with 1 ml of cold acetone at –20°C and centrifuged at 15 000 × *g* for 10 min. The resulting protein pellet was resuspended in 30 μl of water, 3 μl of BME and 30 μl of SDS-PAGE sample buffer (Laemmli, 1970), mixed, and incubated at 70°C for 10 min.

The SDS-PAGE buffers and gels were prepared according to Laemmli (1970). The stacking gels were 5% T, 2.6% C, and the resolving gels were 15% T, 2.6% C 1.5 mm thick. 18 cm×16 cm gels were run in Hoeffer SE 660 apparatus at 20 mA until the dye reached the bottom edge of the gel. After electrophoresis, the gels were silver stained using the procedure of Gromova and Celis (2006).

### SKCS and Total Protein Analysis

The grain hardness, weight, moisture, and diameter of 50 kernels were measured using the Single Kernel Characterization System (SKCS) 4100 (Perten Instruments) for each individual kernel.

The total grain protein content was determined by the Dumas method (Dumas, 1831). The samples of 250 mg of ground seeds in three replicates were combusted at 960°C in the presence of oxygen, followed by afterburning at 800°C, and dried with P_2_O_5_. The resultant nitrogen oxide was reduced to nitrogen. The content of nitrogen in the probes was recounted for crude protein using Rapid N Cube (Elementar, Germany). The formula *N* × 6.25 was applied.

### Statistical Analysis

Statistical analysis was performed using MS Excel and R studio^3^. Obtained data were analyzed by Pearson correlation or one way analysis of variance. Samples that did not meet the ANOVA assumptions were tested by Kruskal–Wallis test.

## Results

### Artificial microRNA Cassette Specification

**FIGURE 1:**
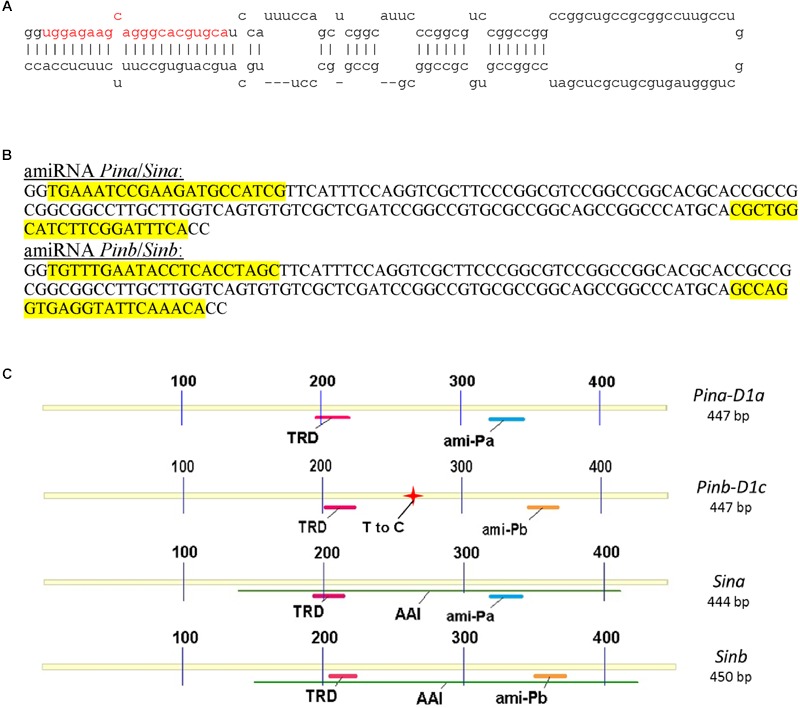
**Construction of artificial miRNA for silencing of *Pin* and *Sin* genes and schematic diagram of *Pin* and *Sin* coding sequences.** Secondary structure of the wild type of the Tae-miR164 precursor **(A)**. Sequence of the modified precursor **(B)**. Schematic diagram of silenced genes with important domains and matching *Pina*-derived and *Pinb*-derived amiR fragments **(C)**. TRD, tryptophan rich domain; AAI, alpha-amylase inhibitor; T to C, thymine to cytosine transition at position 266 (leucine to proline change in position 60 of *Pinb-D1c* allele compared to wild *Pinb-D1a*).

Artificial microRNA for silencing of *Pina* or *Sina* genes (*Pina*-derived amiR) and *Pinb* or *Sinb* genes (*Pinb*-derived amiR) was constructed on the base on precursor miRNA of wheat *Tae-miR164* (Mirbase.org: MI0006173) (**Figure 1A**). The final sequence of *Pina*-derived amiR and *Pinb*-derived amiR cassettes with the highlighted 21 nt precursors is presented in **Figure 1B**. These specific precursors matched the *Pina* and *Pinb* or *Sina* and *Sinb* sequences as shown in **Figure 1C**.

### Transformation Efficiency is Dependent on the amiR Sequence

Potentially transgenic plants were selected on hygromycin. The transgenic character of these plants was proven by PCR amplification of T-DNA with two pairs of specific primers.

Transgenic plants were obtained after transformation of two cultivars of wheat (cv. Kontesa and cv. Torka) with the *Pinb*-derived amiR vector. Transformation efficiency was 2.97 and 0.09%, respectively (**Table 1**). In contrast, failure to obtain *Pina*-derived amiR transformed plants after inoculation of 2753 immature embryos of three cultivars of wheat in six experiments was observed.

**Table 1:** Transformation efficiency of wheat and triticale cultivars with *Pina*-derived amiR (ami-Pa) and *Pinb*-derived amiR (ami-Pb) vectors.

Cultivar/species	Vector pBract214	Number of transformation experiments	Number of explants	Number of lines	Number of plants	Transformation efficiency (%) (*SE*)
**Wheat**						

Kontesa	ami-Pa	4	2127	0	0	0.00 (0.00)
	ami-Pb	1	640	19	28	2.97 (^∗^)
Torka	ami-Pa	1	411	0	0	0.00 (^∗^)
	ami-Pb	2	1158	1	1	0.09 (^∗^)
Ostka	ami-Pa	1	215	0	0	0.00 (^∗^)

**Triticale**						

Wanad	ami-Pa	5	4287	3	3	0.07 (0.04)
	ami-Pb	2	1033	39	54	3.78 (0.32)

*^∗^, not applicable; *SE*, standard error.*

Similar results were obtained for triticale cv. Wanad. Transformation efficiency was high (3.78%; ±0.32) with the *Pinb*-derived amiR cassette and very low (0.07%; ±0.04%) with the *Pina*-derived amiR cassette after inoculation of 4287 immature embryos in five independent experiments.

### Silencing of *Pinb* and *Sinb* Genes by *Pinb*-Derived amiR Is Highly Efficient in T_1_ Generation and Triggers Simultaneous Silencing of *Pina*/*Sina*

**FIGURE 2:**
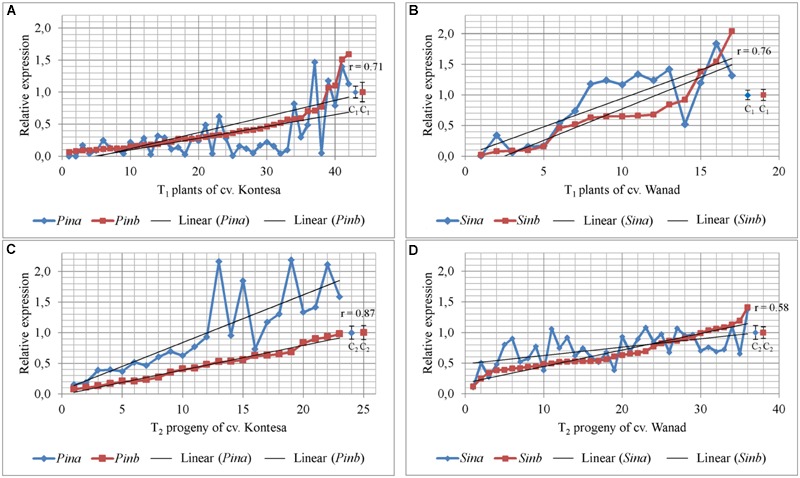
**Relative expression of *Pin* and *Sin* genes in 26 DAP kernels of T_1_ transgenics and their T_2_ progeny silenced with *Pina*-derived and *Pinb*-derived amiR constructs.** Values for *Pina* and *Pinb* genes in T_1_ silenced plants **(A)** and T_2_ progeny **(C)** of wheat cv. Kontesa. Relative expression of *Sina* and *Sinb* genes in T_1_ silenced plants **(B)** and T_2_ progeny **(D)** of triticale cv. Wanad. C, control lines; *r* = positive and significantly important (α = 0.01) correlation between *Pina* and *Pinb*, and *Sina* and *Sinb*.

Segregation ratio of PCR positive (PCR+) to PCR negative (PCR-) plants in 17 T_1_ lines of wheat and 12 T_1_ lines of triticale indicated that 2/3 of these lines were one-locus transgenics and the remaining 1/3 were two-loci transgenics. Relative expression of both *Pina* and *Pinb* or *Sina* and *Sinb* in 21 DAP kernels silenced with *Pinb*-derived amiR was estimated in 42 transgenic T_1_ plants from 13 T_0_ lines of wheat cv. Kontesa and 18 transgenic T_1_ plants from 12 T_0_ lines of triticale cv. Wanad (**Figures 2A,B**). Ten of 13 tested wheat lines were one-locus and three lines were two-loci transgenics. In case of triticale 10 lines were one-locus and two lines were two-loci transgenics. Relative expression of *Pinb* in T_1_ wheat plants, ranging from 0.06 up to 1.59, was below 0.2 in 11 plants (26%) from seven lines and between 0.2 to 0.5 in 20 plants (48%) from 10 lines. All plants with relative expression equal or lower then 0.8 compared to control set to 1.0 (±0.19) are considered as “silenced,” while relative expression over 1.2 indicated overexpression.

Similar results of downregulation in the *Pinb*-derived amiR transgenic plants were obtained for the second, *Pina* gene. The same 31 (74%) of T_1_ plants showing relative *Pinb* silencing below 0.5 also showed *Pina* silencing at a corresponding or even lower level (**Figure 2A**). The correlation coefficient between silencing of *Pinb* and *Pina* in T_1_ wheat *Pinb*-derived amiR transgenics was high and statistically significant (ρ = 0.71; α = 0.01).

These data of *Pinb* (and *Pina*) gene(s) silencing correlated with expression of amiR specific to the *Pinb* gene. The level of *Pinb*-derived amiR in seven plants with strong silencing of both *Pina* and *Pinb* was from 0.3 × 10^6^ to 68 × 10^6^ fold higher compared to the calibrator sample (i.e., the sample with the lowest detectable amiR level set to 1.0). In another set of seven plants with weaker silencing the values, ranging between 3 × 10^2^ and 7 × 10^2^, were from three to five orders of magnitude lower. The highest level of expression of amiR was found in one-locus transgenic plants and lower level was in two-loci plants.

Relative expression of *Sinb* in T_1_ triticale plants ranged from 0.02 up to 2.04 (**Figure 2B**). Five of them showed very low expression of *Sinb*, below 0.2, with a corresponding low expression of *Sina*. Relative expression of *Sinb* for half of the T_1_ triticale plants ranged from 0.46 to 0.92, but the corresponding *Sina* expression for most of them was above 1.0. Correlation between the expression of *Sinb* and *Sina* in T_1_ was statistically significant (*r* = 0.76; α = 0.01). The expression of *Pinb* and *Pina*, and *Sinb* and *Sina*, was not decreased in very few T_1_ plants of both Triticeae species.

### The Strong Effect of Silencing of *Pin* and *Sin* Genes in the T_1_ Is Not Transmitted to the T_2_ Generation

The transgenic character of all tested T_2_ plants was proved by PCR analysis with specific primers. Relative expression of *Pinb* in T_2_ progeny of wheat cv. Kontesa ranged from 0.08 to 0.98 and for *Pina* from 0.15 to 2.16 (**Figure 2C**). About half of the plants out of 23 tested showed relative *Pinb* expression below 0.5. The *Pina* expression in the same plants was considerably higher. Relative expression for *Pina* in plants with 0.5–1.0 values of *Pinb* silencing was very unstable and in most of the progeny ranged from 1.0 to 2.2. The correlation between *Pinb* and *Pina* expression in T_2_ (**Figure 2C**) was strong, positive, and statistically significant (*r* = 0.81; α = 0.01).

**FIGURE 3:**
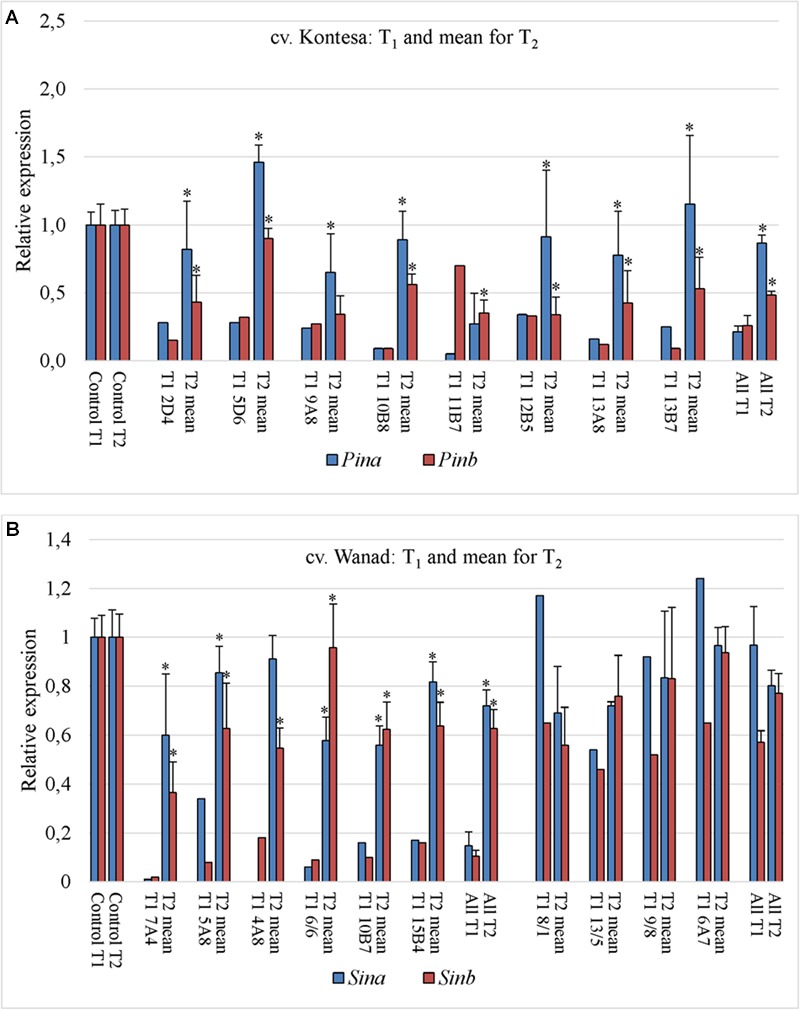
**Transmission of silencing effect from T_1_ to T_2_ generation in two polyploid cereal species.** Relative expression of *Pin* and *Sin* genes in T_1_ silent plants and mean for their T_2_ progeny of wheat cv. Kontesa **(A)** and triticale cv. Wanad **(B)**; ^∗^ – significantly different from T_1_.

The relative expression of both genes silent in eight tested T_1_ plants of wheat was much higher in their T_2_ progeny comparing to T_1_ (**Figure 3A**). The significance of difference was confirmed by Kruskall–Wallis test (*p* = 2.41e-05, α = 0.05). The mean expression value for *Pinb* in T_1_ plants was 0.26 (±0.07) and for *Pina* was 0.21 (±0.04). The mean values for both *Pinb* and *Pina* genes in T_2_ progeny were respectively 0.48 (±0.07) and 0.87 (±0.12). The values in the T_2_ progeny were higher and more variable for *Pina* compared to the silenced *Pinb*. The mean relative expression for *Pinb* in T_2_ was higher in individual lines as well as in all lines compared with T_1_. The values for *Pinb* were less variable than for *Pina* in the T_2_ generation. The data of very weak or lack of *Pinb* and *Pina* silencing in T_2_ were correlated with the level of significantly weak or lack of *Pinb*-derived amiR, which ranged from 0 to 3.55 × 10^3^ (compared to 0.3 × 10^6^ to 68 × 10^6^ in T_1_).

Relative expression of *Sinb* in T_2_ progeny of triticale cv. Wanad ranged from 0.12 to 1.41 and for *Sina* from 0.11 to 1.38 (**Figure 2D**). About ¼ ; of the progeny out of 36 tested showed relative *Sinb* silencing up to 0.5. For half of the progeny *Sinb* relative expression was from 0.5 to 1.0 and for the rest above 1. The correlation between *Sinb* and *Sina* expression in T_2_ (**Figure 2D**) was positive and statistically significant (*r* = -0.58; α = 0.01). The relative expression of *Sinb* and *Sina* in the T_2_ progeny of seven silenced in T_1_ transgenic plants of triticale increased significantly (Kruskall–Wallis: *p* = 4.844e-05, α = 0.05) (**Figure 3B**). The mean value for *Sinb* in T_1_ plants was 0.11 (±0.02) and for *Sina* was 0.15 (±0.06). The mean values for both *Sinb* and *Sina* genes in the T_2_ progeny were respectively 0.63 (±0.08) and 0.72 (±0.06). Otherwise these values were at a similar level (around 0.6–1.0) in T_2_ progeny of non-silenced or weakly silenced T_1_ plants. As in the case of *Pinb* and *Pina* in wheat, the values of very weak or lack of *Sinb* and *Sina* silencing in T_2_ correlated with very weak or lack of relative expression of *Pinb*-derived amiR (from 0 to 13-fold compared to calibrator sample).

**FIGURE 4:**
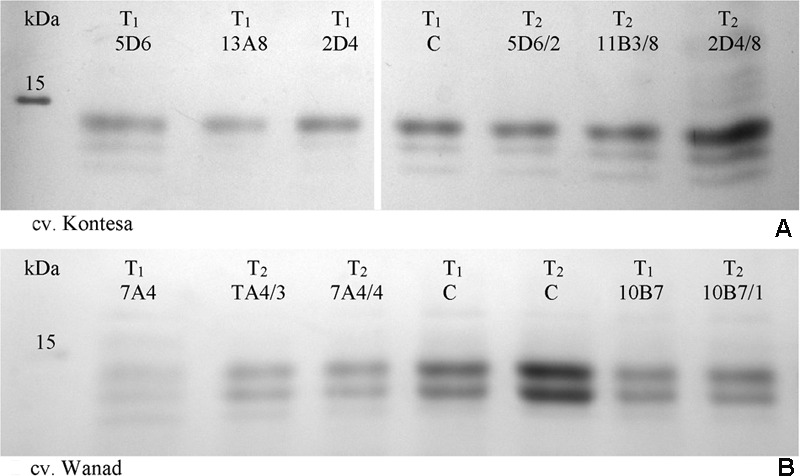
**SDS-PAGE fractionation of mature seed starch-associated proteins isolated from water washed starch from T_1_ and T_2_ progeny of wheat cv. Kontesa (A)** and triticale cv. Wanad **(B)**. C, control lines from T_1_ and T_2_ generations; M, molecular weight marker.

The data of very low in T_1_ or similar to control in T_2_ transcript level of the *Pina*/*Pinb* genes in wheat and the *Sina*/*Sinb* genes in triticale were correlated with very low or similar to control level of *puroindoline a*/*puroindoline b* or *secaloindoline a*/*secaloindoline b* (**Figures 4A,B**).

### Silencing of *Pin* and *Sin* Genes in T_1_ Determines Grain Hardness

**FIGURE 5:**
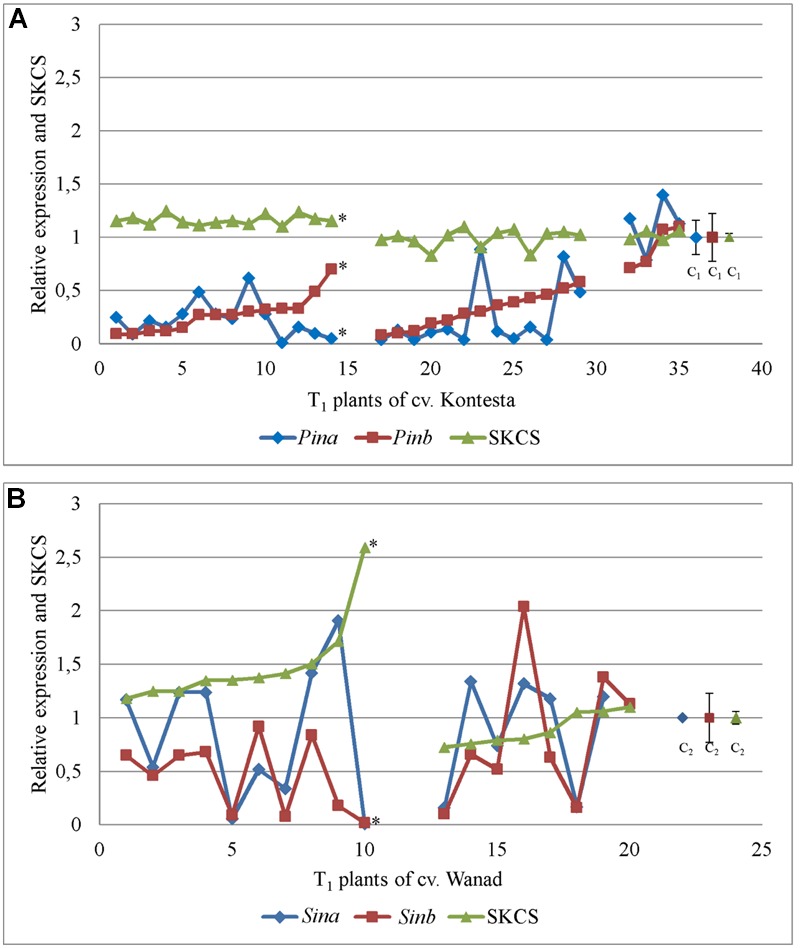
**Relative expression of *Pin* (A)** and *Sin* **(B)** genes in 26 DAP kernels of T_1_ plants silenced with *Pinb*-derived amiR versus hardness index [Single Kernel Characterization System (SKCS)] of their grains (T_2_ progeny); ^∗^ – the correlation between *Pina* or *Pinb* expression and SKCS in 1–14 plants of cv. Kontesa and between *Sinb* expression and SKCS in 1–10 plants of cv. Wanad was negative and significantly important at α = 0.05.

There were two main groups of T_1_ plants with silent *Pin* and *Sin* genes in both tested Triticeae species. In the first one, the significantly lower expression of *Pin* genes in wheat cv. Kontesa and *Sinb* gene in triticale cv. Wanad were associated with increased grain hardness index, measured by the SKCS (Kruskall–Wallis for *Pina*/SKCS: *p* = 2.287e-08, α = 0.05; for *Pinb*/SKCS: *p* = 5.422e-09, α = 0.05; for *Sina*/SKCS: *p* = 0.1136, α = 0.05; and for *Sinb*/SKCS: *p* = 0.0009204, α = 0.05). In the second group, the relative values of SKCS in the T_1_ silent transgenics were not changed in wheat cv. Kontesa or were slightly lower in triticale cv. Wanad (**Figure 5**). Correlation coefficients between these two groups of wheat were 0.289 and 0.294 and for the two groups of triticale were 0.522 and 0.517 and were not significant.

The mean SKCS of the grains collected from all tested 46 lines of the T_1_ generation of wheat was 8% higher (not significant as confirmed by ANOVA: *p* = 0.109684, α = 0.05) than in control plants, and the highest value was 124% of the control (**Table 2**). The mean SKCS in the grains from 28 tested T_1_ lines of triticale was 30% higher than in the control (not significant as confirmed by Kruskall–Wallis: *p* = 0.2159, α = 0.05). The highest SKCS was 260% of the control.

**Table 2:** Range and means of hardness index [Single Kernel Characterization System (SKCS)] and other parameters of grains collected from silent T_1_ lines of wheat cv. Kontesa and triticale cv. Wanad.

Cultivar /vector/	No. of lines	SKCS		Weight (mg) (*SE*)	Moisture (%) (*SE*)	Diameter (mm) (*SE*)
		Range	Mean (*SE*)			
***cv. Kontesa***						

/amiPb/	46	56.57–84.69	73.57 (0.95)	39.47 (0.53)	8.00 (0.45)	3.05 (0.13)
control	5	59.60–74.10	68.07 (2.48)	42.15 (0.65)	8.19 (0.49)	3.04 (0.10)

***cv. Wanad***						

/amiPb/	28	9.23–66.62	32.83 (2.67)	43.13 (1.40)	8.44 (0.48)	2.92 (0.14)
control	4	21.25–27.08	25.20 (1.05)	47.89 (3.34)	9.10 (0.54)	3.00 (0.13)

The mean SKCS in the grains collected from all tested 46 transgenic lines of the T_2_ generation of wheat was 5% higher than in control lines, and from 28 tested T_2_ lines of triticale was 9% higher than in control.

### Total Protein Content Is Decreased in Wheat and Increased in Triticale Silenced Plants

Mean total protein content in the same grains used for SKCS from silent 46 T_1_ lines of wheat cv. Kontesa was 14.65% (±0.23) and was significantly lower than in the control (16.40% ± 0.35), which was confirmed by ANOVA (*p* = 0.037; α = 0.05) (**Table 3**). Opposite data were obtained for triticale. Mean total protein content in the grains from 28 silent T_1_ was 13.74% (±0.48) and was higher than in control (12.81% ± 0.30), but statistically not significant.

**Table 3:** Range and means of total protein content in the grains collected from silent T_1_ lines of wheat cv. Kontesa and triticale cv. Wanad.

Cultivar /vector/	No. of lines	Protein (%)		Protein/dry weight (%)	
		Range	Mean (*SE*)	Range	Mean (*SE*)
**Kontesa**					

/amiPb/	46	10.09–17.86	14.65 (0.23)	11.86–19.58	16.27 (0.35)^∗^
control	5	13.03–18.33	16.40 (0.91)	14.13–20.06	17.92 (1.03)^∗^

**Wanad**					

/amiPb/	28	9.23–18.22	13.74 (0.48)	10.09–20.19	15.17 (0.53)
Control	4	12.15–13.58	12.81 (0.30)	13.47–15.06	14.17 (0.30)

*^∗^Differences statistically significant (ANOVA: *p* = 0.037; α = 0.05).*

**FIGURE 6:**
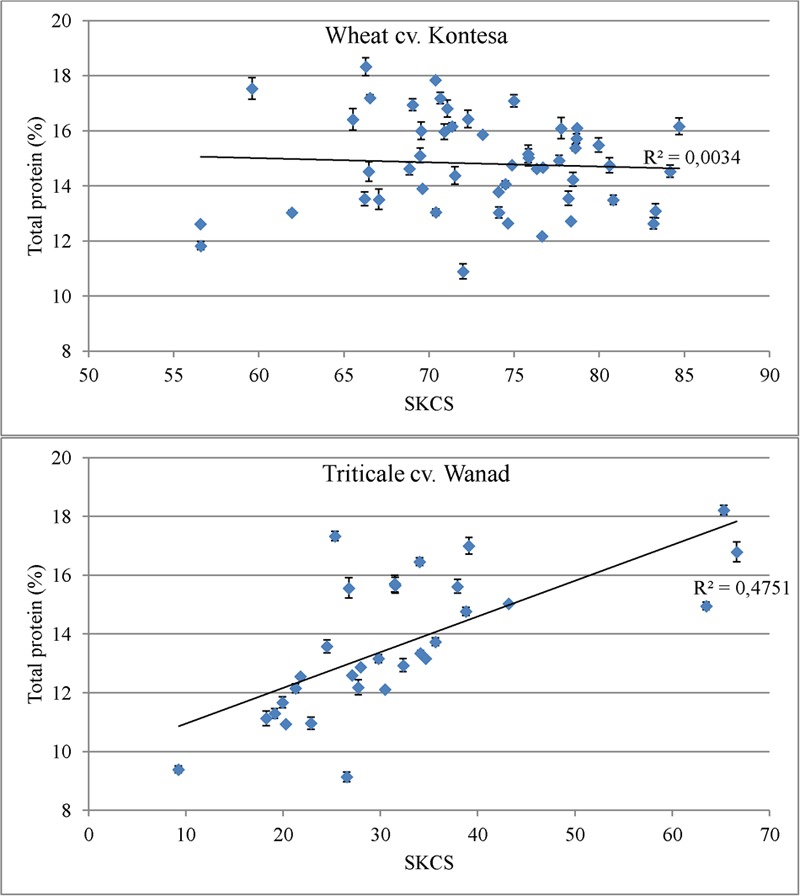
**Linear regression analysis between total protein content (%) and grain hardness (SKCS) in wheat cv. Kontesa and triticale cv. Wanad**.

There was no correlation between grain hardness index and total protein in the T_2_ grains from T_1_ plants with silenced *Pina* and *Pinb* (*r* = -0.05848) (**Figure 6**). However, a strong positive, statistically significant correlation between hardness index and total protein in triticale transgenics was observed (*r* = 0.68925; α = 0.05). Total protein content in triticale cv. Wanad was increasing together with the increasing values of grain hardness.

## Discussion

The technology of gene silencing by amiRNAs exploits endogenous miRNA precursors to generate short, 21 nt fragments of RNAs which are complementary to the target mRNA of the gene to direct gene silencing (Ossowski et al., 2008). In this research, a silencing cassette was designed based on the endogenous precursor of wheat miRNA *Tae-miR164*. The precursor was cloned in wheat by Yao et al. (2007). The main criteria for choosing this precursor were that the sequence of miRNA is conserved and the expression of miRNA is strong, constitutive and not tissue specific. miR164 targets NAC transcription factors and is conserved among dicot and monocot plants. We have also confirmed strong expression of miR164 in various wheat organs (roots, leaves, inflorescences, seeds) at different developmental stages (Gasparis et al. unpublished).

Native fragments of miRNA of the precursor were replaced with the 21 nt fragments complementary to the coding sequences of *Pin* and *Sin* genes chosen for silencing. This kind of strategy of designing amiRNAs by replacing the sequences of miRNA within the miRNA precursor without disrupting its structural features was originally proposed by Schwab et al. (2006). The WMD3 platform^4^ used to design the specific 21 nt fragments from *Pin* and *Sin* genes has a built-in algorithm which allowed to predict the optimal sequence of *microRNA* for the target genes. Such designing should provide high efficiency of silencing, specific to the cultivars of the two polyploid cereal species used in the experiments.

The *Pina*- and *Pinb*-derived amiR silencing cassettes were introduced into the wheat and triticale by *Agrobacterium*-mediated transformation. This method of genetic transformation is the method of choice since we documented that the biolistic approach might stimulate strong off-target effects in the case of gene silencing by siRNAs (Zalewski et al., 2012). The similar negative effect of biolistic transformation compared to the *Agrobacterium*-mediated method was documented in the case of transgene expression in cereals (Dai et al., 2001; Travella et al., 2006). Unexpectedly, transformation efficiency was found to be dependent on the amiR cassette. Highly efficient transformation in both polyploid species, wheat and triticale, was observed in the case of *Pinb*-derived amiR and no or single transgenic plants were obtained in these species after transformation with the *Pina*-derived amiR cassette despite large numbers of embryos attempted and several repeats of experiments. In our opinion the chosen fragment of amiR for silencing of *Pina* and *Sina* genes might be lethal at the level of development of somatic embryos and/or plantlets, what will be further investigated.

However, we were able to obtain transgenic plants of wheat and triticale after applying the hpRNA type of cassette containing the full coding sequence of *Pina* or *Sina*, respectively (Gasparis et al., 2011, 2013).

Silencing of *Pinb* and *Sinb* in the T_1_ generation of wheat and triticale transgenic plants was highly efficient, attaining a maximum 94 and 98% decrease of gene expression, respectively. A high level of silencing (more than 50%) was obtained for most of the 60 T_1_ plants tested in both species. Moreover, *Pinb* or *Sinb* gene silencing simultaneously decreased the expression of the second *Pina* or *Sina* gene, respectively, showing a strong, positive correlation. The level of silencing of *Pinb* or *Pina* and *Sinb* or *Sina* by siRNAs was not so high in the T_1_ generation of wheat and triticale, reaching over an 80% decrease of gene expression in single segregating plants (Gasparis et al., 2011, 2013). In those experiments, the effect of silencing was increased when silencing cassettes for both genes were used in cotransformation experiments. Similar to our results the impact of down-regulation of *SBEIIb* in rice by amiRNA- and by hairpin RNA-mediated silencing has been reported (Butardo et al., 2011). The target gene expression was decreased much more in amiRNA silenced lines than in siRNA silenced lines.

Likewise, in the case of amiR, the silencing of one of the *Pin* or *Sin* genes simultaneously decreased the expression of the corresponding paralogue. Both wild-type *Pin* genes and wild-type puroindoline (PIN) proteins are required to determine soft-grain texture and mutation in either or both genes results in hard wheat (reviewed in Bhave and Morris, 2008; Nadolska-Orczyk et al., 2009; Pauly et al., 2013). The two-event model of co-operative action of both puroindolines explains why some of SNP-type of mutation may alter grain texture (Alfred et al., 2014). It is possible that this co-operative action of both proteins influence co-operative expression of both genes.

Although the *Pinb*-derived amiR silencing cassette is stably inherited in wheat and triticale, unexpectedly the effect of silencing of both *Pin* and both *Sin* genes by amiRs in most of the cases was not transmitted to the T_2_ generation or tended to decrease. Compatible with this lack of transmission of the silencing effect is the lack or very low expression of these amiRs in T_2_. To explain this, we are going to perform bisulfite sequencing of the amiRNA construct regions to identify methylation status affecting transcript level of the insert over the generations. The only data on the effect of amiR silencing in the T_2_ or later generations of cereal species were documented by Butardo et al. (2011) and Fahim et al. (2012). The expression of *SBEIIb* silenced by amiRNAs revealed more than a fivefold decrease in the homozygous T_3_ generation, and two T_4_ lines had a stably down-regulated SBEIIb level and in one line the protein was almost undetectable (Butardo et al., 2011). In the case of *WSMV* virus resistance the inheritance of immunity was confirmed in the T_2_ generation (Fahim et al., 2012). The same *Pin* genes in wheat and *Sin* genes in triticale silenced by siRNAs using hpRNAi types of cassettes were stably down-regulated by over 90% in T_2_ to T_4_ and displayed the predicted, hard grain phenotype up to the T_4_ generation (Gasparis et al., 2011, 2013).

A high level of silencing of *Pin* and *Sin* genes and a corresponding high level of expression of *Pinb*-derived amiRs in T_1_ have been correlated with decreased content of puroindoline or secaloindoline proteins and increased grain hardness. All T_1_ plants of wheat tested were on average 8% harder than control lines, and the highest value was 124% of the control. The values of the hardness index were especially high for triticale lines typically representing soft grain species. The mean was 30% higher than in the control, and the highest hardness index was 2.6 times higher than in control lines. The silencing of wheat *Pin* genes by *Pinb*-derived amiR in T_1_ lines had a stronger effect on grain hardness than in the case of siRNAs (Gasparis et al., 2011). Moreover, the hardness index in the case of silencing of triticale *Sin* genes by *Pinb*-derived amiR was especially high, contrary to the lack of change of this index after using the siRNA (Gasparis et al., 2013). Therefore we might suppose that in the case of triticale the silencing of *Sin* genes by siRNA was not sufficiently specific to determine grain hardness. In conclusion, we might state that silencing effects of *Pin* genes in allopolyploid wheat and especially *Sin* genes in allopolyploid triticale prove the high specificity of the amiRs, underlined also by others for diploid cereals (Warthmann et al., 2008; Carbonell et al., 2015). In the case of *SBEIIb* silencing in rice the amiRNA technique was more effective in producing more extreme starch properties than the hp-RNA technique (Butardo et al., 2011).

Silencing of *Pin* genes in wheat by amiRNAs correlated with decreased puroindoline content and increased grain hardness, which caused significant decrease of total protein content. The silencing of *Sin* genes in triticale by amiRNAs resulted in decreased secaloindoline content and spectacular increase of grain hardness. Contrary to the results in wheat, the triticale grain hardness positively correlated with total protein content. Opposite trends were observed for total protein content in grains of both species, when *Pin* and *Sin* genes were silenced with the siRNA type of RNAi cassettes (Gasparis et al., 2011, 2013). In the case of wheat, total protein content was increased, but it decreased in triticale. These discrepancies might be the result of different ways of silencing of *Pin* and *Sin* genes in both species and again underline the specificity of these two technologies.

## Conclusion

We proved that amiRNA technology is highly efficient for silencing of endogenous, structural genes in allopolyploid cereal species, wheat and triticale. However, the main limitation might be stability of the silencing effect through generations. Silent T_1_ lines accumulated high levels of amiRNAs, which caused strong down-regulation of expression of the target genes. Moreover, the silencing was highly specific, displaying perfectly the predicted phenotypes in both species, induced by repression of the target genes. Although the transgenes/silencing cassettes were stably inherited through generations, the high expression of amiRNAs as well as the silencing effect was in most of the cases not transmitted to the T_2_ generation or in other tended to decrease. This result, different from some diploid cereals, might be dependent on more complex regulation of gene expression in allopolyploid species including endogenous miRNA activity. The effect of lack of inheritance of the silenced phenotype over the T_1_ generation differs from silencing results of the same hardness genes by siRNAs, where the level of down-regulation of target genes and the expected phenotype were proved up to the T_4_ generation. Summarizing both techniques, RNAi silencing by amiRNAs using specific precursor miRNA or by siRNAs with the hpRNAi type of cassettes efficiently down-regulated the target genes; however, amiRNAs are more specific, but the silencing signal might be interrupted through the generations. Otherwise, the effect of silencing by siRNAs is stably inherited. Both techniques might be used for analysis of gene function in species with large and complex genomes, but only hpRNAi can be applied to obtain stable inherited lines with desired characteristics. A big advantage of both techniques is the possibility of obtaining silent lines differing in the level of silencing, which might not be achieved by other techniques such as gene editing.

## Author Contributions

SG designed and performed most of the experiments, MK and MP performed selected experiments and statistical analysis, WO coordination and codiscussion, AN-O conception and design, analysis and interpretation, and wrote the manuscript.

## Conflict of Interest Statement

The authors declare that the research was conducted in the absence of any commercial or financial relationships that could be construed as a potential conflict of interest.

## Footnotes

1 http://wmd3.weigelworld.org

2 https://www.jic.ac.uk/technologies/crop-transformation-bract/

3 https://www.rstudio.com/

4 http://wmd3.weigelworld.org
